# Remotely Supervised, Home-Based Transcranial Direct Current Stimulation for Major Depressive Disorder: Systematic Review and Meta-Analysis

**DOI:** 10.2196/92522

**Published:** 2026-09-04

**Authors:** Motti Haimi, Natália Almeida-Antunes, Simao Pedro Rodrigues Ferreira, Nuno Barbosa Rocha

**Affiliations:** 1 Health Systems Management Department, The Max Stern Yezreel Valley College, D.N Emek Yezreel, Mizra, Northern District, 1930600, Israel, 972 04-642-3423; 2 The Ruth and Bruce Faculty of Medicine, Technion – Israel Institute of Technology, Haifa, Israel; 3 RISE-Health, Center for Translational Health and Medical Biotechnology (TBIO), ESS, Technical University of Porto, Porto, Portugal

**Keywords:** transcranial direct current stimulation, major depressive disorder, systematic review, meta-analysis, telemedicine, remote supervision, home-based treatment, brain stimulation

## Abstract

**Background:**

Major depressive disorder affects over 280 million people worldwide, and access to effective treatment remains limited. Transcranial direct current stimulation (tDCS) is a noninvasive option, and portable devices now allow for home-based delivery under varying degrees of remote supervision.

**Objective:**

This study aimed to systematically review and meta-analyze the efficacy, safety, feasibility, and acceptability of home-based and remotely supervised tDCS for depressive disorders.

**Methods:**

Following the PRISMA (Preferred Reporting Items for Systematic Reviews and Meta-Analyses) 2020 and PRISMA-S (Preferred Reporting Items for Systematic Reviews and Meta-Analyses literature search extension) guidelines, we searched MEDLINE, Embase, Web of Science, the Cochrane databases, ClinicalTrials.gov, and the World Health Organization International Clinical Trials Registry Platform up to July 2025, with backward and forward citation searching. Two reviewers independently screened records, extracted data, and assessed risk of bias (version 2 of the Cochrane risk-of-bias tool for randomized trials, Newcastle-Ottawa Scale for observational studies, and Critical Appraisal Skills Programme for qualitative studies) and certainty of evidence (Grading of Recommendations Assessment, Development, and Evaluation; GRADE).

**Results:**

This review included 12 distinct studies (16 reports), of which 6 (50%) were randomized sham-controlled trials forming the meta-analytic pool. Active home-based tDCS produced a small, statistically significant improvement over sham (pooled Hedges *g*=0.36, 95% CI 0.06-0.66; *P*=.03; *I*^2^=34.3%). The effect was not robust to removal of the single largest positive trial (omitting the one study from 2025: *g*=0.39, 95% CI −0.12 to 0.91), and trial-level results were mixed: the 2 largest trials (one unsupervised [n=210] and one self-administered [n=141]) were negative on their primary depression outcomes, whereas the largest real-time supervised trial (n=174) was positive (between-group 95% CI 0.51‐4.01; *P*=.01). This estimate was concordant in direction with an independent peer-reviewed meta-analysis of overlapping trials, which reported a pooled Montgomery-Åsberg Depression Rating Scale reduction (weighted mean difference −2.74, 95% CI −4.19 to −1.29) and Hamilton Depression Rating Scale reduction (weighted mean difference −2.24, 95% CI −4.16 to −1.49), attenuating to nonsignificance (*P*>.05) in major depressive disorder without comorbid cognitive impairment. The pooled effect fell at or near the minimal clinically important difference. GRADE certainty was moderate. Adverse events were predominantly mild: one pilot study was terminated early for skin lesions, and one nonfatal suicide attempt occurred in an unsupervised trial.

**Conclusions:**

Home-based and remotely supervised tDCS produces a small, statistically significant but clinically modest antidepressant effect that is sensitive to the inclusion of the largest positive trial, with the 2 largest trials being negative. The available controlled evidence does not establish supervision intensity as a determinant of efficacy. Current data are insufficient to recommend routine clinical adoption; adequately powered trials with standardized supervision and longer follow-up are needed.

## Introduction

Major depressive disorder (MDD) affects more than 280 million people worldwide and is a leading cause of disability [1]. Despite pharmacological and psychotherapeutic options [2,3], roughly 30% to 50% of patients do not achieve adequate response to first-line interventions [4], and access to care is constrained by cost, travel, and limited specialist availability. These gaps have motivated interest in noninvasive brain stimulation and in particular transcranial direct current stimulation (tDCS) [5-7].

tDCS delivers a weak direct current (typically 1‐2 mA) through scalp electrodes, producing polarity-dependent shifts in cortical excitability rather than triggering action potentials [5,8,9]. In depression, the conventional montage places the anode over the left dorsolateral prefrontal cortex (DLPFC) and the cathode over the right DLPFC or right supraorbital area, targeting prefrontal circuitry implicated in mood regulation [10]. Repeated sessions are thought to induce longer-lasting plasticity-like changes. A detailed account of the molecular and network mechanisms is beyond the scope of this review; the consideration most relevant here is that therapeutic engagement depends on consistent stimulation parameters and accurate electrode placement, which become central when treatment moves from the clinic to the home.

Clinic-based tDCS has shown modest antidepressant efficacy in meta-analyses, with effect sizes generally in the small to moderate range [6,11-14]. However, conventional clinic protocols typically require 10 to 20 supervised visits over several weeks [14-16], with associated travel costs, time commitments, limited specialist availability, and reduced access in rural or underserved areas. Portable devices and remote monitoring platforms have enabled home-based delivery, with the potential to improve access while preserving therapeutic standards [17,18]. The COVID-19 pandemic accelerated acceptance of remote health care delivery. Home-based tDCS protocols differ in supervision intensity, ranging from real-time video oversight to asynchronous monitoring to fully self-administered use [7,16,19,20], and this variation may bear on efficacy, safety, and feasibility.

Previous systematic reviews have focused primarily on clinic-based tDCS [21-24], and the reviews that have addressed home-based delivery have been limited by small sample sizes, heterogeneous protocols, and limited attention to implementation factors [25-27]. A peer-reviewed meta-analysis of home-based tDCS randomized trials [28] reported small, statistically significant reductions on clinician-rated depression scales and concluded that routine clinical use could not yet be recommended. The present review provides an independent quantitative synthesis and is broader in scope: alongside the meta-analysis, it synthesizes evidence on feasibility, supervision models, qualitative acceptability, bipolar depression, treatment resistance, and older adult populations, situating the modest efficacy signal within the practical requirements for safe home delivery. Our aims were to (1) quantify the efficacy of home-based tDCS vs sham in randomized trials; (2) characterize its safety profile; (3) evaluate implementation feasibility, including training, supervision models, and technology platform performance; and (4) assess patient acceptability.

## Methods

### Protocol Registration and Reporting Standards

This systematic review was prospectively registered with PROSPERO (registration number CRD420251109275; [29]) and is reported according to the PRISMA (Preferred Reporting Items for Systematic Reviews and Meta-Analyses) 2020 statement [30] and the PRISMA-S (Preferred Reporting Items for Systematic Reviews and Meta-Analyses literature search extension) [31]. The completed PRISMA 2020 checklist is provided in Checklist 1. Where a checklist item was not part of the methodology, this is stated in the text. Post hoc analyses are labeled as exploratory.

### Eligibility Criteria

We included studies of adults (aged ≥18 years) with MDD, bipolar depression, or clinically significant depressive symptoms diagnosed via standardized criteria (*Diagnostic and Statistical Manual of Mental Disorders, 4th* and *5th Edition*, and *International Classification of Diseases, 10th Revision*) or validated rating scales. Eligible interventions were tDCS delivered in home-based, remotely supervised, or self-administered settings with any stimulation parameters, electrode montage, or adjunctive treatments. Eligible comparators were sham tDCS, waitlist, treatment as usual, or active comparators; uncontrolled pretest-posttest studies were eligible for feasibility and safety outcomes only. Eligible designs were randomized controlled trials, nonrandomized controlled trials, cohort studies, case series with 5 or more participants, feasibility studies, and qualitative studies of patient experience. We excluded exclusively clinic-based tDCS, pediatric populations, case reports or series with fewer than 5 participants, studies not reporting depression outcomes, animal studies, and conference abstracts without full text availability. The review was restricted to direct current stimulation; transcranial alternating current stimulation and other transcranial electrical stimulation modalities were not eligible.

### Information Sources and Search Strategy

We searched MEDLINE (Ovid) from 1946 to June 2025; Embase from 1974 to July 2025; the Web of Science Core Collection from inception to July 2025; and the CENTRAL and the Cochrane Database of Systematic Reviews, ClinicalTrials.gov, and the World Health Organization International Clinical Trials Registry Platform up to July 15, 2025. The strategy combined 3 concept groups (intervention, setting, and condition) using Boolean operators, with database-specific controlled vocabulary (MeSH for MEDLINE and Emtree for Embase), free-text terms, and field modifiers. Complete database-specific search strings are provided in Multimedia Appendix 1. We performed backward citation searching of included studies and prior reviews and forward citation searching of included studies and screened relevant trial registry entries.

### Study Selection and Data Extraction

Two reviewers independently screened all 363 deduplicated records by title and abstract. The reviewers disagreed on 3% (n=11) of the records (raw agreement: n=352, 97%; Cohen κ≈0.78, indicating substantial agreement), and all disagreements were resolved through discussion or consultation with a third reviewer. We used a piloted standardized extraction form covering study characteristics, population, intervention details (supervision model, training protocol, device, stimulation parameters, and duration), comparator, outcomes, adverse events, feasibility, and acceptability. Where a single trial generated multiple publications, the trial was counted once, and its reports were linked so that no participant contributed to a pooled estimate more than once. Corresponding authors were contacted for missing or unclear data.

### Risk of Bias and Certainty of Evidence

Randomized trials were assessed using version 2 of the Cochrane risk-of-bias tool for randomized trials [32], observational studies were assessed using the Newcastle-Ottawa Scale [33], and qualitative studies were assessed using the Critical Appraisal Skills Programme qualitative checklist. Certainty of evidence for primary outcomes was rated using the Grading of Recommendations Assessment, Development, and Evaluation (GRADE) framework [34].

### Synthesis Methods

Randomized sham-controlled trials reporting continuous depression outcomes were pooled. Because the included trials reported outcomes on different scales (Hamilton Depression Rating Scale [HAM-D] and 17-item HAM-D [HDRS-17] [35], Montgomery-Åsberg Depression Rating Scale [MADRS] [36], and Beck Depression Inventory [BDI] and BDI-II), effects were standardized to the Hedges *g*, with positive values indicating greater improvement under active than under sham stimulation. Trials reporting per-arm means and SDs were entered directly [37-39]; trials reporting standardized between-group effect sizes were entered by converting the reported effect size and its CI to the Hedges *g* with a corresponding SE and pooled by generic inverse variance [25,40,41]. The 3-arm trial by Lee et al [38] was entered as 2 active vs sham contrasts (1 mA vs sham and 2 mA vs sham), with the shared sham group divided between the 2 contrasts to avoid double counting; consequently, these 6 trials contributed 7 independent comparisons to the pooled model. For the study by Oh et al [39], which was null on clinician-rated scales and positive only on the self-rated BDI, the BDI outcome was used, and this was noted as a limitation. Trials were combined using a random-effects model with restricted maximum likelihood estimation of τ^2^ and the Hartung-Knapp-Sidik-Jonkman adjustment [42] implemented in R (version 4.x; R Foundation for Statistical Computing) using the *meta* and *metafor* packages. Heterogeneity was quantified using *I*^2^, τ^2^, and the *Q* test. A prediction interval was not reported: the estimated between-study variance was negligible (τ^2^<0.0001), and with only 6 trials (7 comparisons), the resulting interval was unstable and not meaningfully interpretable. Prespecified sensitivity analyses included leave-one-out estimation and exclusion of the prematurely terminated pilot by Kumpf et al [25]. Subgroup observations examined supervision model, treatment duration, population, and region; because the supervision category was confounded across studies with sample size, country, population, duration, and cointerventions, supervision-related differences were interpreted as hypothesis generating. Small-study effects could not be formally assessed given the small number of trials; funnel plot asymmetry, where examinable, has causes other than publication bias, including clinical and methodological heterogeneity, trial quality, and chance [43]. The independently published estimates of a concordant peer-reviewed meta-analysis [28] are reported alongside our own as external corroboration. Studies not amenable to quantitative synthesis were synthesized narratively; qualitative studies underwent thematic synthesis [44], with overlap between datasets from a single cohort stated explicitly.

## Results

### Study Selection

**Figure 1.**
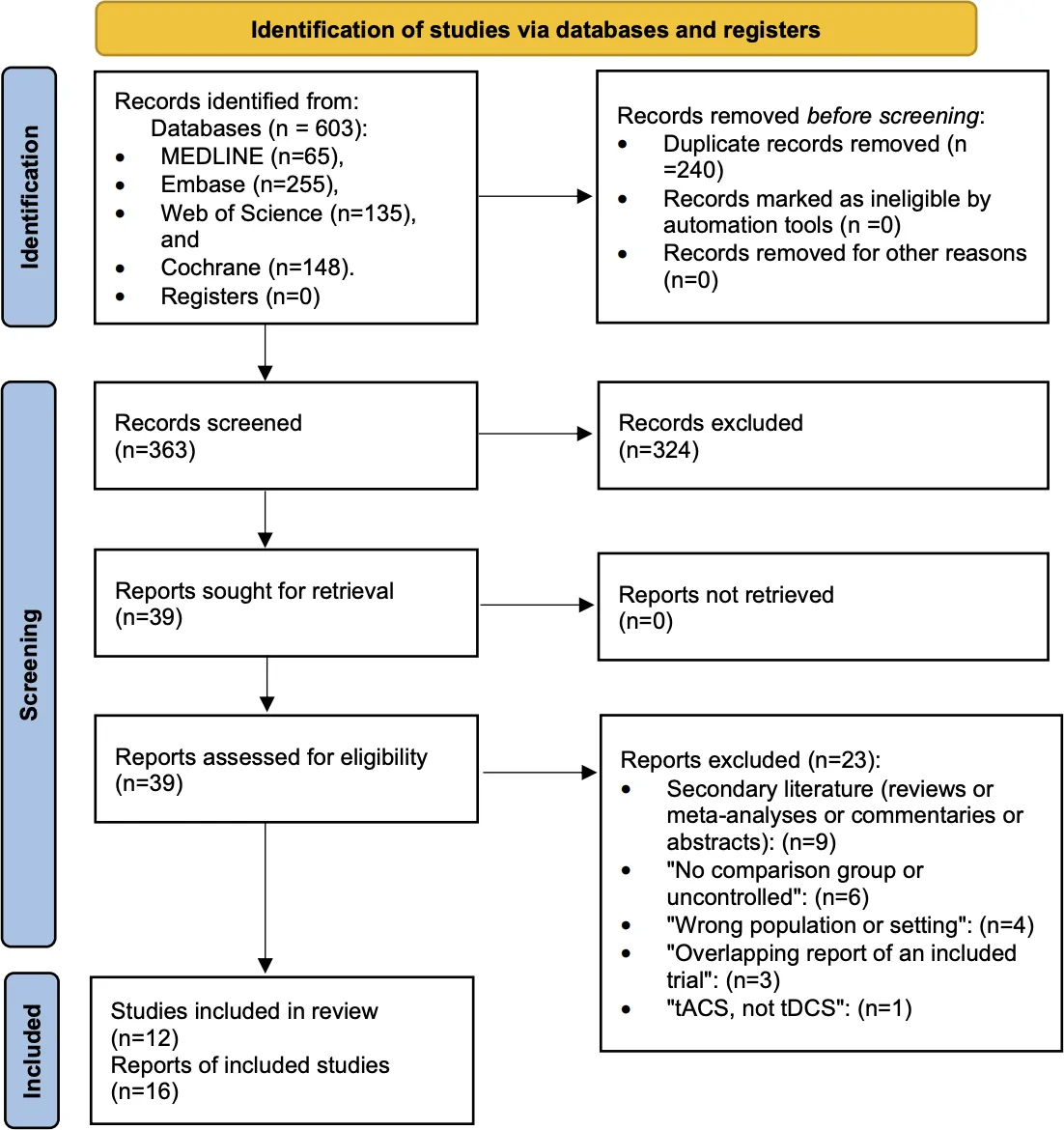
PRISMA (Preferred Reporting Items for Systematic Reviews and Meta-Analyses) 2020 flow diagram. tACS: transcranial alternating current stimulation; tDCS: transcranial direct current stimulation.

The database search identified 603 records (MEDLINE: n=65, 10.8%; Embase: n=255, 42.3%; Web of Science: n=135, 22.4%; Cochrane databases: n=148, 24.5%). Of these 603 records, after removal of 240 (39.8%) duplicates at the identification stage, 363 (60.2%) unique records were screened by title and abstract, of which 324 (89.3%) were excluded. The remaining 39 reports were assessed for full-text eligibility, of which 23 (59%) were excluded due to being secondary literature (reviews, meta-analyses, commentaries, or conference abstracts; n=9, 39.1%); having ineligible designs without a comparison group, including single-arm and case-series reports (n=6, 26.1%); having the wrong populations or settings (n=4, 17.4%); being reports overlapping with an already included trial (n=3, 13%); and using transcranial alternating rather than direct current stimulation (n=1, 4.3%). The review included 16 reports corresponding to 12 distinct studies, of which 6 (50%) were randomized sham-controlled trials forming the meta-analytic pool (Figure 1).

### Study Characteristics

**Figure 2.**
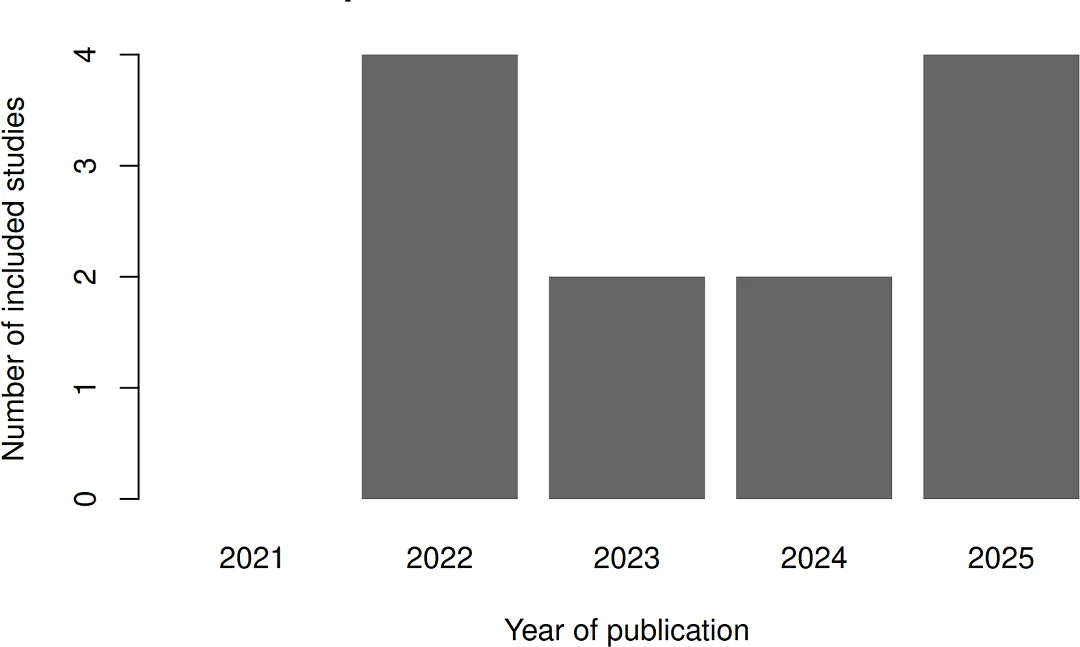
Temporal distribution of the included reports.

Included studies were published between 2021 and 2025 and were conducted in the United Kingdom, the United States, Brazil, South Korea, Germany, the Netherlands, Türkiye, and Australia, as well as through multinational collaborations. Sample sizes ranged from 11 to 210 participants per trial. Supervision ranged from real-time video oversight to asynchronous monitoring to self-administered use. Detailed characteristics, with each study assigned to one design category, one supervision category, and one geographic region, are summarized in Table 1. The trial reported in *Nature Medicine* [37] was the primary publication of a single multisite real-time supervised trial that also generated a preprint, a 6-month follow-up [45], and an electroencephalography substudy [46]; these reports describe 1 trial and contributed once to the pool. The home use trial reported in *JAMA Psychiatry* [40] and its ancillary symptom cluster analysis [47] likewise describe 1 trial. The temporal distribution of the included reports is shown in Figure 2.

**Table 1.** Characteristics of the included studies (12 distinct studies; 16 reports).

Study	Design	Sample size, n	Population	Supervision type	Country	Role in this review
Woodham et al [37], 2025	RCT^a^; double blind; sham	174	MDD^b^	Real time	UK^c^ and US^d^	Meta-analysis
Borrione et al [40], 2024	RCT; double blind; sham; 3 arms	210	MDD	Unsupervised	Brazil	Meta-analysis
Lee et al [38], 2025	RCT; double blind; sham; 3 arms	141	MDD	Self-administered	South Korea	Meta-analysis
Oh et al [39], 2022	RCT; single blind; sham	58	MDD	Self-administered	South Korea	Meta-analysis
Aktürk et al [41], 2025	RCT; single blind; sham	20	MDD	Asynchronous	Türkiye	Meta-analysis
Kumpf et al [25], 2023	RCT; double blind; sham; terminated early	11	MDD	Home based	Germany	Meta-analysis (caveated)
Koutsomitros et al [17], 2023	Controlled; nonrandomized	40	Depression	Asynchronous	The Netherlands	Narrative
Woodham et al [48], 2022	Open label; single arm	26	MDD	Real time	UK	Narrative
Le et al [49], 2022	Case series	16	TRD^e^	Real time	Australia	Narrative
Ruffini et al [50], 2024	Open-label pilot; multichannel	35	MDD	Real time	Multination (US and EU^f^)	Narrative
Cappon et al [51], 2022	Feasibility	Small	MDD (older adults)	Real time	US	Narrative
Lee et al [52], 2022	RCT; double blind; sham	64	Bipolar	Home based	South Korea	Narrative (bipolar disorders)

a RCT: randomized controlled trial.

b MDD: major depressive disorder.

c UK: United Kingdom.

d US: United States.

e TRD: treatment-resistant depression.

f EU: European Union.

### Risk of Bias

Risk of bias among the 6 randomized trials was mixed. The 2 largest adequately powered trials were the most rigorously conducted: the real-time supervised trial [37] and the unsupervised home use trial [40] used computer-generated randomization with allocation concealment, double-blind sham control with verified blinding, and near-complete outcome data and were judged to be at low risk of bias overall. The remaining trials raised some concerns. The multisite trial by Lee et al [38] was double blinded with balanced baselines but documented partial unblinding by week 6 and approximately one-third attrition managed through last observation carried forward imputation. The self-administered trial by Oh et al [39] was single blinded, with its positive result confined to a self-rated outcome, and showed higher attrition in the active arm. The small asynchronous trial by Aktürk et al [41] was single blinded with a small sample and a baseline severity imbalance between arms, and its primary outcome was self-rated. The terminated HomeDC pilot by Kumpf et al [25] was at high risk of bias given its very small sample and early termination for safety. Observational and feasibility studies, assessed using the Newcastle-Ottawa Scale, were predominantly of moderate quality, and the 2 qualitative reports met most Critical Appraisal Skills Programme criteria. Formal assessment of small-study effects (funnel plot and Egger test) was not performed because the small number of trials rendered such tests underpowered and unreliable; the influence of individual trials on the pooled estimate was instead examined through the leave-one-out analysis reported above.

### Primary Efficacy

**Figure 3.**
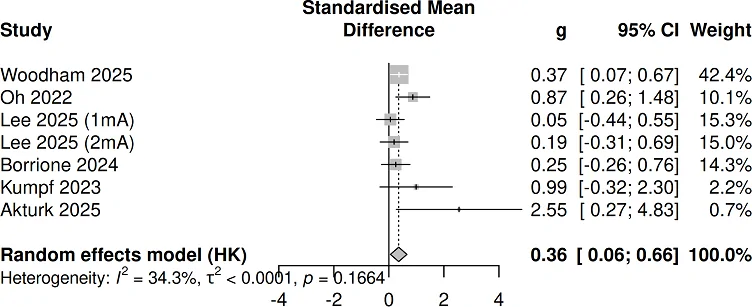
Forest plot of the 6 randomized sham-controlled trials (7 comparisons; random-effects pooled Hedges *g* with 95% CIs) [25,37-41,52]. HK: Hartung-Knapp.

Six randomized sham-controlled trials, contributing 7 independent comparisons (the 3-arm trial by Lee et al [38] provided 2 active vs sham contrasts sharing a common sham group), were pooled in the efficacy meta-analysis (total n≈600 participants): those by Woodham et al [37] (n=174; real-time supervised trial), Borrione et al [40] (n=210; unsupervised), Lee et al [38] (n=141; self-administered), Oh et al [39] (n=58; n=45 analyzed; self-administered), and Aktürk et al [41] (n=20; asynchronous) and the terminated HomeDC pilot (Kumpf et al [25]; n=11; Table 2). Pooled across these comparisons using a random-effects model with the Hartung-Knapp-Sidik-Jonkman adjustment, active home-based tDCS produced a small, statistically significant improvement over sham (Hedges *g*=0.36, 95% CI 0.06-0.66; *P*=.03) with moderate heterogeneity (*I*^2^=34.3%; τ^2^<0.0001; Figure 3 [25,37-41,52]).

**Table 2.** Per-trial outcomes for the 6 randomized sham-controlled trials.

Trial	Sample size (active/sham), n	Primary depression scale	Active vs sham result	Direction
Woodham et al [37], 2025	87/87	HDRS-17^a^	Mean improvement 9.41, SD 6.25 points for the active group vs mean 7.14, SD 6.10 points for the sham group; between-group 95% CI 0.51-4.01 (*P*=.01); response: 58.3% vs 37.8% (*P*=.02)	Positive
Borrione et al [40], 2024	137/73	HDRS-17	No significant group difference; Cohen *d*=−0.25 to +0.05; *P*>.30 in all cases	Negative
Lee et al [38], 2025	96/45	HAM-D^b^	No significant group difference at week 6 (change: mean 6.96, SD 6.82 for the sham group; mean 7.40, SD 8.51 for the 1-mA group; mean 8.59, SD 9.18 for the 2-mA group; *F*=0.497; *P*=.61)	Negative
Oh et al [39], 2022	20/25 (analyzed)	HAM-D, MADRS^c^, and BDI^d^	HAM-D: *F*=0.04 and *P*=.85; MADRS: *F*=2.37 and *P*=.13; BDI: pretest-posttest mean change −10.40, SD 7.98 vs −3.80, SD 7.01, *t*=2.95, and *P*<.05	Self-report only
Aktürk et al [41], 2025	11/9	BDI-II	Significant active advantage (Cohen *d*=−2.55, 95% CI −4.83 to −0.27); adherence: mean 14.45 of 15 sessions	Positive (small)
Kumpf et al [25], 2023	~5/~5	MADRS and HAM-D	Symptoms declined; active arm not superior (Cohen *d*=−0.99); terminated for skin lesions	Negative or inconclusive

a HDRS-17: 17-item Hamilton Depression Rating Scale.

b HAM-D: Hamilton Depression Rating Scale.

c MADRS: Montgomery-Åsberg Depression Rating Scale.

d BDI: Beck Depression Inventory.

This pooled effect was not robust to the influence of the single largest positive trial. In leave-one-out analysis, omitting the study by Woodham et al [37] rendered the estimate nonsignificant (*g*=0.39, 95% CI −0.12 to 0.91; *P*=.11), whereas omitting the smallest trials left it essentially unchanged (omitting the study by Aktürk et al [41]: *g*=0.34, 95% CI 0.07-0.61, with *I*^2^ falling to 9.8%; omitting the study by Kumpf et al [25]: *g*=0.34, 95% CI 0.01-0.67). The reduction in heterogeneity on removal of the smallest trial indicates that the dispersion in the pool was driven largely by the small high-effect trials rather than by the larger trials.

Trial-level results were mixed and consistent with this pattern. In the largest real-time supervised trial (n=174) [37], active tDCS produced a significantly greater improvement on the HDRS-17 than sham (mean improvement of 9.41, SD 6.25 points in the active arm vs 7.14, SD 6.10 points in the sham arm; between-group 95% CI 0.51‐4.01; *P*=.01), with a clinical response rate of 58.3% vs 37.8% (*P*=.02). The 2 next-largest trials found no significant difference from sham on depressive outcomes: the unsupervised home use trial (n=210) [40] reported no significant between-arm differences on the HDRS-17 (Cohen *d*=−0.25 to +0.05), and the multisite self-administered trial (n=141) [38] reported no significant group difference on HAM-D change at week 6 (mean 6.96, SD 6.82 in the sham arm; mean 7.40, SD 8.51 in the 1-mA arm; mean 8.59, SD 9.18 in the 2-mA arm; *F*_2_=0.497; *P*=.61). The self-administered trial by Oh et al [39] (n=58) showed a significant active vs sham advantage on the self-rated BDI (pretest-posttest change: mean −10.40, SD 7.98 vs mean −3.80, SD 7.01; *t*_43_=2.95; *P*<.05) but no significant difference on the clinician-rated HAM-D (*F*=0.04; *P*=.85) or MADRS (*F*=2.37; *P*=.13). The small asynchronous trial (n=20) [41] showed a significant active advantage on the BDI-II (Cohen *d*=−2.55, 95% CI −4.83 to −0.27), with high adherence. The terminated HomeDC pilot (n=11) [25] showed symptom decline over time without superiority of active over sham (Cohen *d*=−0.99).

This estimate was concordant in direction and magnitude with an independent peer-reviewed meta-analysis of the overlapping home-based randomized trials [30], which reported a small pooled reduction in the MADRS (weighted mean difference [WMD] −2.74, 95% CI −4.19 to −1.29; *I*^2^=0%) and the HAM-D (WMD −2.24, 95% CI −4.16 to −1.49; *I*^2^=47%), attenuating to nonsignificance when restricted to MDD without comorbid cognitive impairment (WMD −2.32, 95% CI −4.72 to 0.07; *P*=.06). Our pooled *g* of 0.36 lies in the small-effect range and corresponds to a between-group difference at or near the minimal clinically important difference. GRADE certainty was moderate for the primary efficacy outcomes (Table 3). For external benchmarking, a clinic-based individual patient data meta-analysis [11] reported response rates of 30.9% vs 18.9% (number needed to treat=9), a direction broadly compatible with the home-based evidence, although no claim of superiority over standard treatments was supported.

**Table 3.** Grading of Recommendations Assessment, Development, and Evaluation (GRADE) summary of findings—home-based transcranial direct current stimulation vs sham for depression^a^.

Outcome	Trials, n	Pooled effect	Heterogeneity (*I*^2^)	Certainty	Downgrade reason
Depression—pooled; this review	6 (7 comparisons; n≈600 participants)	Hedges *g*=0.36 (95% CI 0.06-0.66)	34.3%	Moderate	Imprecision; few trials
Depression—MADRS^b^ [30]	4 (n=269 participants)	WMD^c^ −2.74 (95% CI −4.19 to −1.29)	0%	Moderate	Imprecision; few trials
Depression—HAM-D^d^ [30]	4 (n=438 participants)	WMD −2.24 (95% CI −4.16 to −1.49)	47%	Moderate	Imprecision; few trials
Depression—HAM-D, MDD^e^ only [30]	3 (n=358 participants)	WMD −2.32 (95% CI −4.72 to 0.07)^f^	61%	Low	Imprecision; inconsistency
Adverse events	6	Predominantly mild; 2 serious signals	—^g^	Low	Heterogeneous definitions; few events

a This review’s pooled Hedges *g* is from a random-effects model with Hartung-Knapp-Sidik-Jonkman adjustment; weighted mean difference rows are the concordant estimates reported by an independent peer-reviewed meta-analysis [30]. Minimal clinically important difference≈3 points for the 17-item Hamilton Depression Rating Scale.

b MADRS: Montgomery-Åsberg Depression Rating Scale.

c WMD: weighted mean difference.

d HAM-D: Hamilton Depression Rating Scale.

e MDD: major depressive disorder.

f *P*=.06.

g Not applicable (not pooled).

### Supervision Model

The 6 controlled trials spanned real-time supervised [37], asynchronous [41], self-administered [38,39], unsupervised [40], and home-based monitored [25] delivery. There were positive trials in the real-time, asynchronous, and self-administered (self-rated) categories; trials showing no significant effect were found in the unsupervised and structured self-administered categories. The moderate pooled heterogeneity (*I*^2^=34.3%) was not consistent with a clean separation into efficacious supervised and inert unsupervised subgroups. Because the supervision category was confounded with sample size, country, population, treatment duration, and cointerventions, and because the number of trials per category was small, supervision intensity is reported as a hypothesis for future controlled evaluation rather than as a demonstrated determinant of efficacy. Testing this hypothesis would require a trial that randomizes supervision intensity directly.

### Safety and Tolerability

Across the included studies, adverse events were predominantly mild, transient, and self-limiting; the most common were skin redness, tingling, mild headache, and localized scalp discomfort (Table 4). Because event definitions, ascertainment, and denominators differed across studies, we report adverse events per study rather than as a pooled odds ratio. In the largest blinded trial [37], the adverse event categories that were significantly more common with active than sham stimulation were skin redness (64% vs 18%, respectively; *P*<.001) and trouble concentrating; other categories did not differ significantly. In the unsupervised trial [40], skin redness and heat or burning sensations were more frequent in the active arms. In the self-administered trial by Oh et al [39], 2 of 20 active participants developed mild electrical burns that resolved after dermatology referral. Serious events were rare: the German HomeDC pilot [25] was terminated early because of accumulated skin lesions in the active arm, which the investigators attributed to insufficient safety monitoring to detect events within an appropriate time frame, and one nonfatal suicide attempt occurred in the unsupervised arm of the largest unsupervised trial, assessed using the Columbia-Suicide Severity Rating Scale [40,53]. No treatment-emergent mania was reported in the bipolar depression studies. Impedance variability was higher in home-based than clinic-based sessions, underscoring the importance of impedance-aware monitoring. These findings indicate a generally favorable but not risk-free safety profile in which adequate training and monitoring of skin condition and impedance are important.

**Table 4.** Adverse events by study.

Study	Most common events	Serious events	Notes
Woodham et al [37], 2025	Skin redness (64% active vs 18% sham; *P*<.001) and trouble concentrating	None	Only redness and concentration differed significantly
Borrione et al [40], 2024	Skin redness and heat or burning (more frequent in the active arm)	One nonfatal suicide attempt (tDCS^a^-only arm)	Largest unsupervised trial
Oh et al [39], 2022	Mild electrical burns (2/20 in the active arm) and one transient headache	None	Burns resolved after dermatology referral
Aktürk et al [41], 2025	Adverse event incidence: 0.63% of sessions	None	High adherence
Kumpf et al [25], 2023	Skin lesions in the active arm	Trial terminated early for safety	Monitoring judged to be insufficient
Lee et al [52], 2022 (bipolar disorders)	Greater pain score in the active arm	No treatment-emergent mania	Completion: 64%

a tDCS: transcranial direct current stimulation.

### Implementation and Feasibility

Feasibility was generally high in the studies that reported it. In the real-time supervised feasibility study [48], 92.3% (24/26) of participants with MDD completed the 6-week protocol (attrition: 7.7%). Under asynchronous supervision, 90% (18/20) of the participants completed all prescribed sessions in one study [17], and adherence averaged 14.45 of 15 sessions in another [41]. In the self-administered trial by Oh et al [39], 77.6% (45/58) of the randomized participants completed the protocol, with discontinuation attributed predominantly to compliance rather than adverse events. In the multichannel telesupervised pilot [50], a high proportion of participants completed the full session schedule. The unsupervised trial [40] reached high procedural completion despite null efficacy, indicating that adherence is necessary but not sufficient for clinical benefit. Reported feasibility metrics, training designs, and denominators varied across studies and are therefore described narratively rather than pooled.

Successful and unsuccessful protocols converged on broadly similar parameters: 2-mA intensity, 30-minute sessions, anode over the left DLPFC and cathode over the right DLPFC or right supraorbital region, and daily or near-daily sessions during the acute phase, with durations from 3 to 10 weeks. Several devices were represented (Flow Neuroscience, Soterix Medical, PlatoScience, Neuroelectrics multichannel systems, and the Ybrain device used in the Korean trials [38,39]); the number of trials per device platform was too small for between-device comparison.

### Patient Acceptability

Acceptability was favorable in the studies that assessed it. In one feasibility study [48], all completing participants rated the intervention as “very acceptable” or “quite acceptable,” and the qualitative reports described convenience, tolerability, and favorable comparison with pharmacotherapy. Acceptability is reported per study using each study’s own measure rather than as a pooled proportion because the instruments and constructs differed. Thematic synthesis of the qualitative studies [54-56] drew on 2 independent datasets—a UK MDD cohort [56] and a UK bipolar depression cohort reported jointly in 2 publications [54,55] from the same research group—and identified recurring themes of perceived helpfulness, tolerability, manageable treatment burden, positive ethical perceptions, gratitude for access to a novel option, and favorable comparison with pharmacotherapy. Because the qualitative evidence was derived from 2 nonindependent UK samples, the breadth of the thematic synthesis is limited, and transferability to other health care systems and cultural contexts cannot be assumed.

### Population-Specific Findings

Five of the 6 controlled trials enrolled adults with unipolar MDD, with the mixed pattern of results described above; the pooled effect was small and not robust to removal of the single largest positive trial. Bipolar depression was addressed by a single home-based randomized trial (Lee et al [52]; n=64), which found no significant active vs sham difference on the HDRS-17 (time-by-group: *F*=2.060; *P*=.11), with no treatment-emergent mania; the supporting open-label and qualitative reports [54,55] were derived from one UK cohort and were uncontrolled. Treatment-resistant depression was represented by a small uncontrolled case series [49]; no randomized sham-controlled trial of home-based tDCS in this population was identified. Feasibility in older adults was supported by tele-supervised pilots [50,51] without controlled efficacy data. Because treatment duration was confounded with supervision model, population, and trial size, no causal inference about a duration-efficacy relationship was drawn. The evidence base was concentrated in high-income health care systems, with no trials from low- and middle-income settings.

## Discussion

### Principal Findings

This systematic review and meta-analysis found that home-based and remotely delivered tDCS produced a small, statistically significant but clinically modest antidepressant effect (Hedges *g*=0.36, 95% CI 0.06-0.66) based on 6 randomized sham-controlled trials and moderate-certainty evidence. The effect lies in the small range and corresponds to a between-group difference at or near the minimal clinically important difference. Critically, it was not robust to the removal of the single largest positive trial: omitting the study by Woodham et al [37] rendered the pooled estimate nonsignificant, indicating that the overall signal leaned substantially on one adequately powered trial whereas the 2 largest trials were negative. Safety was generally favorable, but the studies that had a control group had 2 specific safety signals—early termination of a home-based pilot for accumulated skin lesions and one nonfatal suicide attempt in an unsupervised trial—that warrant structured monitoring. The intervention was well accepted by participants in the studies that measured acceptability.

Our independently computed estimate is concordant in direction and magnitude with that of a peer-reviewed meta-analysis of overlapping trials [28], which likewise reported small, statistically significant reductions on clinician-rated depression scales below thresholds for clinical significance and concluded that routine clinical use cannot yet be recommended. The agreement between 2 analyses using different effect metrics and partially different trial sets strengthens confidence in the qualitative conclusion: a small, fragile, heterogeneity-sensitive effect rather than a robust therapeutic signal. The contribution of the present review is its broader implementation-focused scope, situating the modest efficacy signal within the feasibility, supervision, safety, and acceptability evidence relevant to real-world delivery.

The available controlled evidence does not establish supervision intensity as a determinant of efficacy. Positive trials spanned real-time, asynchronous, and self-administered delivery, whereas trials showing no significant effect included both unsupervised and structured self-administered protocols; the moderate pooled heterogeneity (*I*^2^=34.3%)—driven largely by the smallest trials rather than by supervision category on leave-one-out analysis—is not consistent with a clean split into efficacious supervised and inert unsupervised subgroups. Supervision category was confounded with sample size, country, population, treatment duration, adjunct medication, and recruitment context. Any apparent supervision-outcome association, therefore, reflects an uncontrolled between-trial contrast and is hypothesis generating; it should be tested in a trial that randomizes supervision intensity directly.

The pooled effect of home-based tDCS overlaps with the lower end-of-effect ranges reported for established depression treatments [57,58], but no claim of superiority is supported; number-needed-to-treat figures vary substantially with outcome definition, comparator, and severity threshold. Home-based tDCS may serve as a potential option for selected patients—those seeking nonpharmacological approaches, those with limited access to clinic-based care, or those with medication intolerance—rather than as a clearly superior alternative. The bipolar depression signal is preliminary, resting on one null randomized trial and uncontrolled reports from a single cohort, and warrants dedicated adequately powered trials.

Successful home delivery requires structured training, adequate monitoring to detect the events most likely to occur (impedance excursions and skin changes), and integration into existing services. The early termination of the German pilot indicates that home delivery is not automatically low risk; the same skin lesion category observed at low frequency elsewhere accumulated to an unacceptable rate when monitoring was insufficient. This is an argument for adequate monitoring rather than for clinic-based delivery. Reimbursement and regulatory frameworks should reflect supervision and infrastructure requirements rather than treating home delivery as automatically less expensive or uniformly low risk.

Residual heterogeneity reflects differences across populations, protocol variations within nominal supervision categories, and patient-level factors including neuroplasticity-related genetic polymorphisms (eg, BDNF Val66Met and 5-HTTLPR) that remain unassessed in most trials [59]. Blinding is challenging in home-based tDCS because participants may perceive stimulation sensations, which is a structural limitation of this literature; one included trial documented partial unblinding by the end of treatment. Small-study effects and publication bias could not be formally assessed given the small number of trials, although the leave-one-out analysis showed that the pooled estimate was sensitive to the small high-effect trials. The concentration of trials in high-income settings limits global generalizability.

### Limitations

This review has several limitations. The controlled evidence base was small and dominated by a single large positive trial alongside 2 negative large trials; the pooled estimate was not robust to the removal of that positive trial; heterogeneity in populations, devices, durations, and cointerventions limits pooling; trials were entered using a mix of raw mean and effect size data because not all reported per-arm statistics; the qualitative synthesis rested on 2 nonindependent UK datasets; the bipolar and treatment resistance evidence was uncontrolled or based on a single null trial; long-term and cost-effectiveness data were scarce; and the literature was concentrated in high-income settings.

### Conclusions

Home-based and remotely supervised tDCS produces a small, statistically significant but clinically modest antidepressant effect (Hedges *g*=0.36) based on the available randomized evidence, an effect that was not robust to the removal of the single largest positive trial and that was concordant with an independent published meta-analysis. Two of the 3 largest trials were negative, and the pooled estimate was sensitive to study selection. The available controlled data did not establish supervision intensity as a determinant of efficacy; the apparent supervision-outcome association reflects an uncontrolled between-trial contrast rather than a tested causal effect. The intervention was generally well tolerated, with predominantly mild adverse events, although the controlled literature contained 2 safety signals that argue for adequate structured monitoring regardless of supervision model. Acceptability and feasibility were favorable in the studies that measured them. The certainty of evidence was moderate, and current evidence is insufficient to recommend routine clinical adoption. Adequately powered randomized trials are needed that randomize supervision intensity directly, use standardized clinician-rated and self-report outcomes, extend follow-up beyond 6 months, include low- and middle-income settings, and report adverse events using a standardized instrument. Home-based tDCS remains a plausible option for selected patients within health care systems that can deliver adequate training, monitoring, and clinical integration.

## Supplementary material

10.2196/92522 Multimedia Appendix 1 Full electronic search strategies

10.2196/92522 Checklist 1 PRISMA checklist.

## Abbreviations

BDI: Beck Depression Inventory
DLPFC: dorsolateral prefrontal cortex
GRADE: Grading of Recommendations Assessment, Development, and Evaluation
HAM-D: Hamilton Depression Rating Scale
HDRS-17: 17-item Hamilton Depression Rating Scale
MADRS: Montgomery-Åsberg Depression Rating Scale
MDD: major depressive disorder
PRISMA: Preferred Reporting Items for Systematic Reviews and Meta-Analyses
PRISMA-S: Preferred Reporting Items for Systematic Reviews and Meta-Analyses literature search extension
tDCS: transcranial direct current stimulation
WMD: weighted mean difference
